# The role of MRI in detection and staging of upper urinary tract cancer: a systematic review of the literature

**DOI:** 10.3389/fonc.2026.1563391

**Published:** 2026-02-19

**Authors:** Cécile Manceau, Lucas Bento, Serge Brunelle, Thomas Prudhomme, Anne Sophie Bajeot, Xavier Game, Michel Soulie, Marie Charlotte Delchier, Fatima-Zohra Mokrane, Mathieu Roumiguié

**Affiliations:** 1 Department of Urology and Kidney Transplantation, Montpellier University Hospital, Lapeyronie Hospital, Montpellier, France; 2 Department of Urology, Kidney Transplantation and Andrology, TSA 50032 Rangueil Hospital, Toulouse, France; 3 Department of Radiology, Institut Paoli-Calmettes Cancer Centre, Marseille, France; 4 Department of Radiology, TSA 50032 Rangueil Hospital, Toulouse, France; 5 Department of Urology, Clinique Pasteur, Toulouse, France

**Keywords:** MRI, DWI, ADC, upper urinary tract tumor, upper urinary tract diagnosis, upper urinary tract staging, upper urinary tract prognosis, uro-MRI

## Abstract

**Introduction:**

Upper urinary tract tumors (UUTT) are imprecisely diagnosed. Recent data have shown the benefit of adding systemic treatments to advanced local stage tumors (≥T2). MRI has provided useful information for evaluating the local T stage of urinary bladder tumors, which may be used for UUTT. The objective of this study was to review the literature on the diagnostic and staging capabilities of MRI for UUTT. Additionally, the methods for performing MRI on the UUT were evaluated.

**Methods:**

This review was conducted according to the PRISMA using MEDLINE and EMBASE. All original articles published between January 2000 and February 2024 that investigated the diagnostic and staging performance of MRI in patients with suspected UUTT were included in this study.

**Results:**

Fifteen studies were included, comprising 999 patients, of whom 593 had UUTT. A wide heterogeneity in the sequences was observed. While standard acquisition (T1 weighted + T2 weighted) showed insufficient diagnostic performance, dynamic contrast imaging (DCE) and diffusion weighted imaging (DWI) presented strong diagnostic scores for pooled sensitivity (92.5% and 93.2%), specificity (76.7% and 84.2%), and accuracy of diagnosis (91.1% and 90.1%), respectively. DWI and apparent diffusion coefficient (ADC) seem to be informative for staging and prognostic evaluation.

**Discussion and conclusion:**

MRI has strong potential to enhance UUTT diagnosis and staging. Despite heterogeneous data and limited evidence, the findings of this study suggest that further large multicentric studies should be conducted to better evaluate the diagnostic performance of MRI in UUT, with a predefined standard acquisition compared to the pathology report of radical nephroureterectomy or long-term follow-up by medical imaging. We hope to establish an Upper Urinary Tract Imaging-Reporting and Data System (MRI UUTI-RADS) score for predicting muscle invasion and tumor stage.

**Patient summary:**

We performed a systematic evaluation and summary of all studies published on the diagnostic and staging capabilities of MRI for upper urinary tract tumors. This review showed a lack of evidence data but a strong potential for MRI. A predefined MRI protocol was proposed for future studies.

**Systematic review registration:**

https://www.crd.york.ac.uk/prospero/, identifier CRD42022319265.

## Introduction

Urothelial carcinomas are the sixth most common tumors in developed countries (1). Upper urinary tract tumors (UUTT) account for 5%–10% of all urothelial carcinomas (1). At diagnosis, the disease is invasive in approximately two-thirds of patients (2, 3). The prognosis for UUTT is good for non-infiltrating lesions (<pT2) with >90% 5 years cancer specific survival probability, but diminishes when UUTT invade the muscle wall between 74% and 12% 5 years cancer specific survival probability (pT2–pT4) (2, 4, 5).

The management of UUTT changes according to the tumor progression risk, ranging from endoscopic treatment with renal conservation to nephroureterectomy (6). Given the poor prognosis of invasive tumors, adjuvant platinum-based chemotherapy is recommended (7–10). Although the response to chemotherapy is difficult to predict, recent data suggest the possibility of offering chemotherapy as a neoadjuvant treatment, often as patients are not cisplatin-eligible due to renal function impairment after nephroureterectomy (11, 12). Preoperative assessment of the tumor stage, which is crucial for offering appropriate systemic treatment, remains difficult (5, 13).

Currently, computed tomography urography (CTU) is the reference imaging method, but it requires contrast media injection and creatinine clearance above 30 mL/min (14). CTU has high diagnostic performance in detecting UUTT (92% sensitivity 92% and 95% specificity) (15); however, CTU cannot differentiate a muscle-infiltrating (≥T2) lesion from a non-infiltrating lesion (<T2).

The reliability of endoscopic UUT biopsy in tumor stage evaluation is low, often underestimating the invasion grade with a 43% upgrade rate (16, 17). Importantly, performing a biopsy exposes the patient to higher risks of morbidity and mortality than CTU, such as a higher frequency of bladder recurrence and risks related to general anesthesia (18).

Recent data have reported that MRI provides useful information for evaluating the local T stage of urinary bladder cancer, particularly in differentiating T1stage or lower tumors from others with 85% sensitivity and 90% specificity (19–21). The development of functional non-contrast imaging sequences, such as diffusion-weighted imaging (DWI), which shows *in vivo* water molecular diffusion, and the apparent diffusion coefficient (ADC), which quantifies the extent of water molecule diffusion calculated using various DWI sets with different b-values, provides information about tissue biophysical properties such as cell organization and density, microstructure, and microcirculation (22, 23). These imaging sequences are used to differentiate between benign and malignant lesions (22).

To date, the role of MRI in the diagnosis of UUTT is limited as an alternative to CTU in patients who present with CTU contraindications (allergies or contraindications for radiation or iodinated contrast media) (8, 9). However, given the added value of MRI in bladder cancer, our hypothesis was that MRI could offer a useful preoperative diagnostic and staging tool for UUTT. A preoperative imaging technique that could differentiate T1stages or lower tumors from others would represent a major advance in patient diagnosis, treatment, and follow-up.

The aim of the current study was to review the existing literature on MRI performance for the diagnosis and staging of UUTT. In addition, the methods and sequences for performing MRI on the UUTT were evaluated.

## Materials and methods

### Literature search strategy and study selection

The study protocol was registered in the PROSPERO database (study no. CRD42022319265). A Preferred Reporting Items for Systematic Reviews and Meta-Analyses (PRISMA) 2020 checklist was followed for the study methodology (**Supplementary Table 1**) (24). To review the entire literature published between January 2000 and July 2024, a systematic search of the major reference databases, MEDLINE (PubMed) and EMBASE (Elsevier), was conducted in August 2024. The details of the search terms used for each database are reported in **Supplementary Table 2**.

Covidence software was used for literature management (Covidence Systematic Review Software, Veritas Health Innovation, Melbourne, Australia. Available at www.covidence.org). After duplicate removal, two investigators (CM and LB) working independently assessed all studies after conducting the primary search according to their title and abstract with guidance from the predefined selection criteria. The same investigators made the final selection based on the full-text versions of the studies and the predefined inclusion and exclusion criteria. They independently screened the records for inclusion and were blinded to each other’s decisions. Disagreements were discussed with a third reviewer (MR) and resolved by consensus.

### Inclusion and exclusion criteria

We included studies that analyzed adult patients with suspected UUTT (Population) who underwent MRI for diagnosis, staging, and/or prognosis evaluation (Intervention). Only prospective or retrospective human adult studies published in English between January 2000 and July 2024 were included in this review. Pathological reports after radical nephroureterectomy were considered the reference standard for result comparison. However, studies using CTU, biopsy, and ureteroscopy associated with active follow-up when patients did not have nephroureterectomy were also included (Comparison). We collected all variables that objectively assessed MRI reliability for diagnosis, staging, prognosis, and MRI modalities (Outcomes). Meta-analyses, reviews, letters, meeting abstracts of unpublished trials, case reports, studies with no more than 20 cases, and articles not written in English were excluded.

### Data extraction

Relevant data for each selected article were extracted in a standardized manner (**Supplementary Table 3**).

### Methodological quality: risk of bias and quality of evidence

The risk of bias for all included studies was assessed by two investigators independently (CM and LB). We evaluated the methodological quality applied to four “risk of bias” domains and three “concerns regarding applicability” domains according to the Quality Assessment of Diagnostic Accuracy Studies 2 (QUADAS-2) tool (25). We also used the Methodological Index for Non-Randomized Studies (MINORS) grading score for clinical studies (26). The MINORS score is a validated tool that uses eight graded questions for non-comparative studies.

### Statistical analysis

Analyses were performed on published data only. No extrapolations were performed. Studies with missing data were excluded from the statistical analyses.

The performance of radiologic imaging was pooled separately using a random-effects model. Studies without extractable data were excluded from the meta-regression analysis.

On a study-specific basis, pertinent diagnostic indicators were derived via cross-tabulation, encompassing metrics such as the area under the receiver characteristic curve, optimal threshold values, sensitivity, specificity at the optimal threshold, and enumeration of true-positive, false-negative, false-positive, and true-negative instances. To address instances of zero counts in the studies, a continuity correction of 0.5 was employed during data analysis. A Diagnostic Test Accuracy meta-analysis was conducted to comprehensively evaluate the combined diagnostic efficacy. Univariate descriptive summary statistics were used to compute the sensitivity and specificity per study, along with their corresponding 95% confidence intervals (CIs). These outcomes were graphically depicted using forest plots, providing insights into inter-study variability. A bivariate normal model was applied to logit-transformed pairs of sensitivities and FP rates using the reitsma function from the mada package. This modeling approach facilitated the estimation of sensitivity and FP rates, accompanied by their respective 95% CIs, and the calculation of the area under the receiver operating characteristic curve.

Heterogeneity was assessed using the Tau^2^ and Chi^2^ tests, with a significance level of p <0.1 indicating statistical significance. Additionally, the I^2^ statistic was employed to evaluate clinical heterogeneity, with I^2^ thresholds of <25%, 25%–49%, 50%–75%, and >75% representing low, moderate, high, and very high heterogeneity, respectively.

Analyses were conducted using open-source R software (version 3.6.2; R Foundation for Statistical Computing, Vienna, Austria) (27) with the mada package citation: Doebler P, Holling H. Meta-analysis of diagnostic accuracy with mada. Available online: https://cran.r-project.org/web/packages/mada/vignettes/mada.pdf . 

The graphs were realized using GraphPadPrism version 8.0.0 for Mac, GraphPad Software, San Diego, California USA, www.graphpad.com.

## Results

### Identification and selection of studies

**Figure 1:**
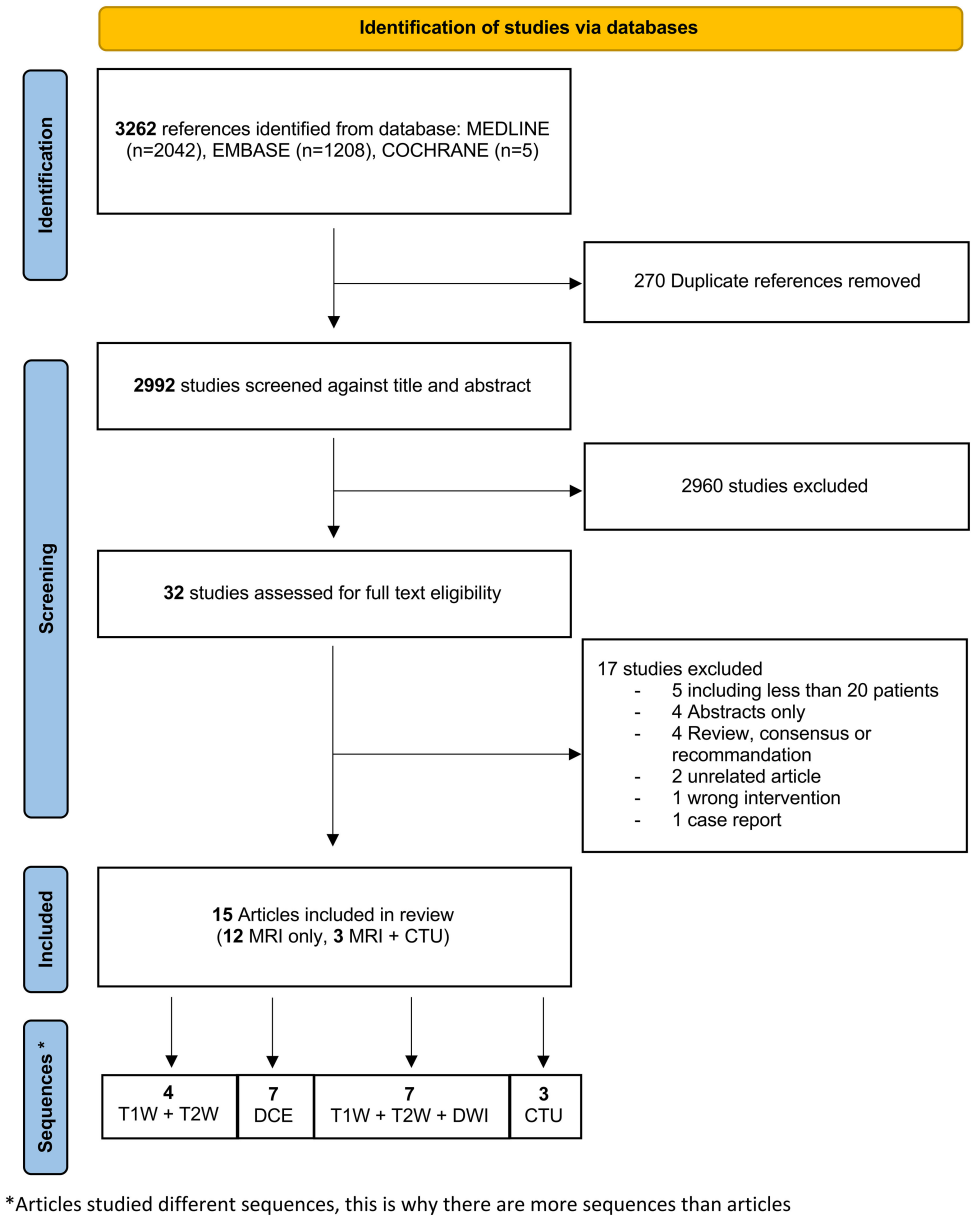
Flowchart of study’s selection. CTU, computed tomography urography; DCE, dynamic contrast enhanced imaging; DWI, diffusion weighted imaging; DCE, dynamic contrast enhanced; T2W, T2 weighted; T1W, T1 weighted.*Articles studied different sequences; this is why there are more sequences than articles.

The PRISMA flowchart is shown in **Figure 1**. Finally, 15 studies were included.

### Studies characteristics

Among the 14 single-center studies and one two-center study, 47% (n = 7) were prospective, while 53% (n = 8) were retrospective (**Table 1**). Fourteen (94%) were published between 2009 and 2018, and only one was published in 2024.

**Table 1:** Characteristics of the studies included.

Study	Objective	Prospective/retrospective	Nb of patient with upper urinary tract tumor/total nb of patient	Sequences	Nb of radiologist (experience)	Comparisons	Reference standard
Akita et al. (2011) (28) Preoperative Tumor Categorization and Prediction of Histopathologic Grading of Urothelial Carcinoma in Renal Pelvis Using DWI	To evaluate the utility of DWI for preoperative T categorization and prediction of the histopathologic grade of renal pelvic cancer. (T3 or Higher Tumors From T2 or Lower Tumors/T3b or Higher Tumors From T3a or Lower Tumors)	Retrospective	N = 40/40	T1W T2W DCE (50 s, 90 s, 120 s, 180 s) DWI(b1,000 s/mm^2^)	2 (15 yr, 11 yr)	T1W + T2W *vs* DCE *vs* T1W + T2W + DWI	Nephroureterectomy pathology
Akita et al. (2018) (29) Performance of DWI post-CT urography for the diagnosis of upper tract urothelial carcinoma: Comparison with selective urine cytology sampling	To evaluate the usefulness of adding DWI to CT urography for diagnosing upper tract urothelial carcinoma.	Retrospective	N = 48/102	T1W T2W DWI(b1,000 s/mm^2^) CTU	2 (30 yr, 15 yr)	T1W + T2W + DWI *vs* CTU	Nephroureterectomy or biopsy pathology, 2 years follow up imaging examination
Lee et al. (2010) (30) Magnetic resonance urography versus retrograde pyelography/ureteroscopy for the exclusion of upper urinary tract malignancy	To evaluate the diagnostic performance of magnetic resonance urography versus retrograde pyelography and/or ureteroscopy in the detection of upper urinary tract neoplasms.	Retrospective	N = 19/35	T1W T2W DCE (20 s, 60 s, 5 min)	1 (not reported)	DCE *vs* ureteroscopy–ureteropyelography	Biopsy, more than 1 year follow up imaging examination
Martingano et al. (2013) (31) Magnetic resonance urography *vs* computed tomography urography in the evaluation of patients with hematuria	To evaluate by direct comparison the image quality of magnetic resonance urography and computed tomography urography and to assess the diagnostic confidence of the two techniques in detecting urothelial malignancy in patients with hematuria	Retrospective	N = 19/35	T1W T2W DCE (10 min) CTU	2 (not reported)	DCE *vs* CTU	Nephroureterectomy or biopsy pathology, follow up imaging examination
Messina et al. (2024) (32) MRI for risk stratification of muscle invasion by upper tract urothelial carcinoma: a feasibility study	To investigate the feasibility of multiparametric MRI to assess the risk of upper tract urothelial carcinoma muscle invasiveness	Prospective	N = 30/30	T1W T2W DCE (2 s, 80 s, 12 min) DWI (b0, 800, 1,000 s/mm^2^) ADC	2 (5yr, 15yr)	MI tumor *vs* NMI tumor	Nephroureterectomy
Roy et al. (2015) (33) DWI in the Etiologic Diagnosis of Excretory Upper Urinary Tract Lesions: Can It Help in Differentiating Benign From Malignant Tumors?	To evaluate the diagnostic performance of high-field DWI in distinguishing benign from malignant lesions of the upper urinary tract	Retrospective	N = 66/98	T1W T2W DWI(b1,000 s/mm^2^) ADC	2 (4 yr, 8 yr)	T1W + T2W + DWI Quantitative ADC	Nephroureterectomy or biopsy pathology, cytology, more than 1 year follow up imaging examination
Shebel et al. (2014) (34) Characterization of upper urinary tract urothelial lesions in patients with gross hematuria using DWI: A prospective study	To evaluate the utility of DWI and ADC values in differentiation between malignant and non malignant lesions of the upper urinary tract in patients with gross hematuria.	Retrospective	N = 23/51	T1W T2W DWI(b800 s/mm^2^) ADC	2 (≥10 yr)	T1W + T2W *vs* T1W + T2W + DWI and ADC	Nephroureterectomy or biopsy pathology, cytology, 3 months clinical follow up
Takahashi et al. (2009) (35) Gadolinium Enhanced Magnetic Resonance Urography for Upper Urinary Tract Malignancy	To evaluate the accuracy of gadolinium enhanced magnetic resonance urography to detect upper urinary tract tumors. Subgroup: surveillance/non surveillance/stent	Retrospective	N = 28/91	T1W T2W DCE (5 min, 10 min)	2 (not reported)	DCE	Nephroureterectomy or biopsy pathology, 1 year follow up imaging examination
Uchida et al. (2014) (36) Diffusion-weighted MRI as a potential imaging biomarker reflecting the metastatic potential of upper urinary tract cancer	To evaluate the role of DWI as an imaging biomarker for upper urinary tract cancer that has already metastasized or will metastasize soon.	Prospective	N = 61/61	T1W T2W DWI(b400 or 800 s/mm^2^)	2 (7 yr, 7 yr)	ADC quantitative for metastatic potential	Nephroureterectomy or biopsy pathology, cytology, more than 1 year follow up imaging examination
Wehrli et al. (2013) (37) Utility of MRI Features in Differentiation of Central Renal Cell Carcinoma and Renal Pelvic Urothelial Carcinoma	To evaluate the utility of various morphologic and quantitative MRI features in differentiating central renal cell carcinoma from renal pelvic urothelial carcinoma	Retrospective	N = 12/60	T1W T2W DWI(b400–800 s/mm^2^) ADC	2 (10 yr, 10 yr)	DWI with ADC and subjective imaging feature to differ renal cell carcinoma from urothelial carcinoma.	Pathologically proven renal urothelial carcinoma and renal cell carcinoma
Wu et al. (2013) (38) Imaging of upper urinary tract cancer: using conventional MRI and diffusion-weighted MRI with different b values	To evaluate the performance of using conventional MRI alone and in combination with DWI with different b values in diagnosis upper urinary tract cancer	Prospective	N = 32/70	T1W T2W DWI (b500 and b1,500 s/mm^2^)	2 (6 yr, 6 yr)	T1W + T2W *vs* T1W + T2W + DWI (b500) *vs* T1W + T2W+ DWI (b1,500)	Nephroureterectomy or biopsy pathology, cytology, more than 18 months follow up imaging examination
Wu et al. (2014) (39) Comparison of computed tomography urography, magnetic resonance urography and the combination of DWI in diagnosis of upper urinary tract cancer	To evaluate the performance of CT Urography, static fluid magnetic resonance urography and combination of CT urography MR urography and diffusion weighted imaging in the diagnosis of upper urinary tract cancer	Prospective	N = 79/163	T1W T2W static fluid MRI CTU	2 (8 yr, 8 yr)	Static fluid MRI *vs* T1W + T2W + DWI *vs* CTU *vs* CTU + DWI *vs* CTU + static fluid MRI +DWI	Nephroureterectomy or biopsy pathology, cytology, more than 18 months follow up imaging examination
Yoshida et al. (2010) (40) Usefulness of DWI in Diagnosis of Upper Urinary Tract Cancer	To prospectively evaluate the diagnostic ability of DWI for detecting upper urinary tract cancer	Prospective	N = 49/76	T1W T2W DCE (30 s, 80 s, 180 s) DWI(b800 s/mm^2^)	2 (4 yr, 4 yr)	T1W + T2W *vs* DCE *vs* T1W + T2W + DWI	Nephroureterectomy or biopsy pathology, 1 year follow up imaging examination
Yoshida et al. (2014) (41) ADC as a prognostic biomarker of upper urinary tract cancer: a preliminary report	To investigate the role of ADC as a biomarker reflecting the aggressiveness of upper urinary tract urothelial cell carcinoma.	Prospective	N = 38/38	T1W T2W DWI (b400 or 800 s/mm^2^) ADC	Not reported	ADC quantitative	Nephroureterectomy pathology
Yoshida et al. (2017) (42) The value of adding DWI for tumor detection and preoperative staging in renal pelvic carcinoma for the reader’s experience	To assess the value of adding DWI or gadolinium-enhanced fat-suppressed T1W to T2W imaging for preoperative T categorization in renal pelvic carcinoma by the reader’s experience using surgical specimens as the reference standard.	Retrospective	N = 49/49	T2W DCE (30 s, 70 s, 180 s) DWI(b800 s/mm^2^)	2 (3 yr, 13 yr)	T2W *vs* T2W + DCE *vs* T2W + DWI	Nephroureterectomy or biopsy pathology

ADC, apparent diffusion coefficient; CT, computed tomography; CTU, computed tomography urography; DCE, dynamic contrast enhanced imaging; DWI, diffusion weighted imaging; MI, muscle invasive; MRI, Magnetic resonance imaging; nb, number; NMI, non-muscle invasive; s, second; T2W, T2 weighted; T1W, T1 weighted; T, Tumor; yr, year.

All studies comprised a total of 999 patients, of which 593 (59.3%) had UUTT, and each study included between 30 and 163 patients, with 20% to 100% presenting UUTT. Among the included studies, 12 (80%) evaluated MRI diagnostic performance, and five (33%) evaluated staging and aggressiveness. Three studies (20%) compared MRI and CTU, and one evaluated the ability of MRI to differentiate central renal cell carcinoma from renal pelvic urothelial carcinoma.

The studies included patients with confirmed UUTT or with a high suspicion of UUTT (gross hematuria or UUT lesions on CTU). The reference standard followed pathology analysis (nephroureterectomy specimen or biopsy) or, if considered inappropriate, ureteropyelogram with 12- or 18-month follow-up.

Studies evaluating the performance of MRI staging have compared ≥T3 tumors with ≤T2 tumors, but only one study has evaluated the ability to identify ≥T2 tumors.

Thirteen (87%) studies had double readings by two independent radiologists. The radiologists had between 3 and 30 years of experience in renal MRI. The number and experience of pathologists were reported in only one study.

### Technical MRI characteristics

MRI was performed using a 1.5T system in 73% (n = 11) of the studies and a 3.0T system in the other studies. T1W and T2W sequences were performed in 93% (n = 14) of the studies, while one study performed T2W sequences only. Contrast enhanced images was acquired in 47% (n = 7) of the studies, consisting of T2W static-fluid acquisition and gadolinium-enhanced T1W sequences (DCE)). Among the studies using contrast enhancement, four performed excretory sequences.

DWI acquisition was performed in 73% (n = 11) of the studies, with varying b-values (400 s/mm^2^, 500 s/mm^2^, 800 s/mm^2^, 1,000 s/mm^2^, and 1,500 s/mm^2^), and 33% (n = 5) of the studies evaluated ADC maps.

### Sequences performances

The MRI sequences used in the included studies were diverse. Baseline sequences such as T2weighted (T2W) and T1weighted (T1W) sequences, were systematically performed. DCE was performed using a contrast media agent with or without late-phase dynamic contrast-enhanced imaging diffusion-weighted imaging. UUT MRI can be improved by adding functional imaging DWI and ADC.

First, we present and analyze the performance of each sequence, and then we compare the sequence performances.

### Baseline sequences T1W and T2W

#### Diagnosis

Four studies evaluated the performance of T1W and T2W (without DCE or DWI) (28, 39, 40, 42) (**Table 2**). Akita et al. (28) and Yoshida et al. (42) evaluated the diagnosis and staging performances exclusively in patients with UUTT. Wu et al. (39) and Yoshida et al. (40) evaluated diagnosis and staging performances in a population with respectively 48% and 64% patients with UUTT.

**Table 2:** Diagnosis performances of MRI for upper urinary tract tumor.

Study	N = (nb cancer//percent cancer) comparison	Sensitivity	Specificity	Accuracy	AUC	Inter observer agreement
T1W + T2W						
Akita et al. (2011) (28)	N = 40 (40//100%) Tumor detection ≤T2 *vs* ≥T3 ≤T3a *vs* ≥T3b	36–34/40 (88%) 14/26 (54%) 13/17 (76%)	9/14 (64%) 17/23 (74%)	23/40 (58%) 30/40 (75%)	0.73 [0.55–0.92] 0.87 [0.72–1.00]	0.69
Wu et al. (2014) (39)	N = 70 (32//46%) Tumor detection	23–24/32 (72%–75%)	23–23/38 (61%)	46–47/70 (66%–67%)		0.80
Yoshida et al. (2010) (40)	N = 76 (49//64%) Tumor detection	31–34/49 (63%–69%)	24–27/27 (89%–100%)	55–61/76 (72%–80%)		0.68
Yoshida et al. (2017) (42)	N = 49 (49//100%) Tumor detection ≤T2 *vs* ≥T3 ≤T3a *vs* ≥T3b	42–44/49 (86%–90%) 69%–73% 60%–75%	82%–84% 95%–96%	73%–78% 73%–86%	0.77–0.79 0.77–0.87	0.429 0.431
CE WITHOUT excretory phase						
Akita et al. (2011) (28)	N = 40 (40//100%) Tumor detection ≤T2 *vs* ≥T3 ≤T3a *vs* ≥T3b	38–38/40 (95%) 17/26 (65%) 15/17 (88%)	11/14 (79%) 18/23 (78%)	28/40 (70%) 33/40 (83%)	0.79 [0.65–0.93] 0.87 [0.80–1.00]	1.0
Yoshida et al. (2010) (40)	N = 53 (38//72%) Tumor detection	33–34/38 (87%–89%)	12–11/15 (80%–73%)	45–45/53 (85%–85%)		0.72
Yoshida et al. (2017) (42)	N = 49 (49//100%) Tumor detection ≤T2 *vs* ≥T3 ≤T3a *vs* ≥T3b	46–46/49 (93%) 78%–76% 83%–86%	82%–75% 100%–100%	80%–76% 92%–94%	0.755–0.755 0.864–0.950	0.427 0.670
CE WITH excretory phase						
Lee et al. (2010) (30)	N = 113 (19//17%) Tumor detection *regions	12/19 (63%)	86/94 (91%)	98/113 (87%)		
Martingano et al. (2013) (31)	N = 35 (29//83%) Tumor detection *regions	83%–86% (a lot of indeterminate segment)	83%–83%	83%–84%	0.938–0.907	0.71
Takahashi et al. (2009) (35)	N = 91 (28//31%) Tumor detection *regions	26–22/35 (74%–62%)	212–211/219 (97%–96%)	238–233/254 (94%–92%)		0.73
T1W + T2W + DWI						
Akita et al. (2011) (28)	N = 40 (40//100%) Tumor detection ≤T2 *vs* ≥T3 ≤T3a *vs* ≥T3b	39–39/40 (98%) 15/26 (58%) 15/17 (88%)	13/14 (93%) 22/23 (96%)	28/40 (70%) 37/40 (93%)	0.79 [0.67–0.91] 0.96 [0.89–1.00]	0.85
Akita et al. (2018) (29)	N = 102 (48//47%) Tumor detection	44–44/48 (96%–96%)	48–49/54 (89%–91%)	92–93/102 (90%–91%)		
Wu et al. (2013) (38)	N = 70 (32//46%) Tumor detection b500 Tumor detection b1,500	29–30/32 (91%–94%) 29–30/32 (91%–94%)	25–26/38 (66%–68%) 32–33/38 (84%–87%)	54–56/70 (77%–80%) 61–63/70 (87%–90%)		0.88 0.86
Yoshida et al. (2010) (40)	N = 76 (49//64%) Tumor detection	45–46/49 (92%–94%)	26–22/27 (96%–81%)	71–68/76 (93%–89%)		0.801
Yoshida et al. (2017) (42)	N = 49 (49//100%) Tumor detection ≤T2 *vs* ≥T3 ≤T3a *vs* ≥T3b	46–46/49 (93%) 74%–80% 81%–83%	77%–79% 93%–100%	76%–80% 88%–92%	0.754–0.796 0.864–0.933	0.755 0.712
CTU						
Akita et al. (2018) (29)	N = 102 (48//47%) Tumor detection	46–47/48 (96%–98%)	42–42/54 (78%–78%)	88–89/102 (86%–87%)		
Martingano et al. (2013) (31)	N = 35 (29//83%) Tumor detection *regions	97%–97% a lot of indeterminate segment	91%–87%	92%–88%	0.994–0.977	0.43
Wu et al. (2014) (39)	N = 163 (79//48%) Tumor detection	75–74/79 (95%–94%)	75–74/84 (89%–88%)	150–148/163 (92%–91%)	0.919–0.912	0.947

ADC, apparent diffusion coefficient; AUC, area under the curve; CTU, computed tomography urography; DWI, diffusion weighted imaging; CE, contrast enhanced imaging; nb, number; imaging, T2W, T2 weighted, T1W, T1 weighted, T, Tumor.

**Figure 2:**
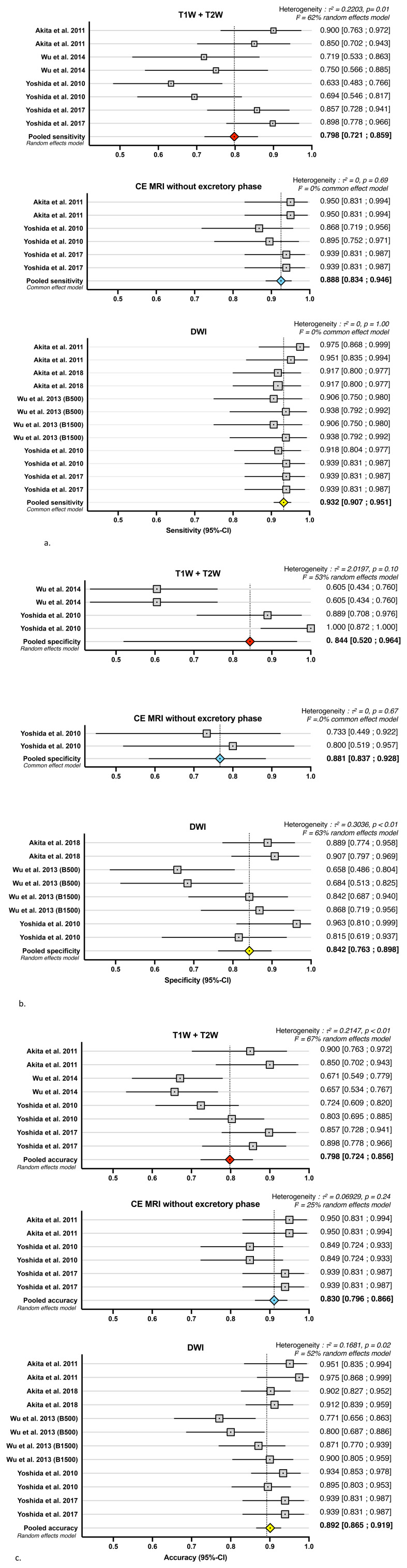
Forest plots of studies included in the meta-analysis show individual and pooled estimates for diagnostic sensitivity **(A)**, specificity **(B)**, and accuracy **(C)**, with 95% confidence interval (CI).

Pooled diagnosis performances showed 79.8% [95% IC 72.1%–85.9%] sensibility, 84.4% [95% IC 52.0%–1.00%] specificity, and 79.8% [95% IC 72.4–85.6%] accuracy (**Figure 2**).

The sensibilities of the different articles were heterogeneous, ranging from 63% to 87%, but the specificities were even more heterogeneous, ranging from 60% to 100% with very wide CIs (**Table 2**).

#### Staging

The staging performance was evaluated by Akita et al. (28) and Yoshida et al. (42). The ≥T3b and ≤T3a tumors discrimination (60% to 76% sensitivity and 74% to 96% specificity) seems to be better than ≥T3 or versus ≤T2 (54% to 73% sensitivity and 64% to 84% specificity) but results are heterogeneous and not statistically significant (**Table 2**) (28, 42). Pooled statistics could not be performed due to non-extractable data.

#### Dynamic contrast enhanced imaging with or without excretory phase

DCE is based on the association of T2W static-fluid acquisition and imaging performed using gadolinium-enhanced T1W sequences (43). Three studies acquired DCE without the excretory phase (28, 40, 42) and performed dynamic contrasted enhanced imaging no later than 180 s after injection; four studies (30–32, 35) acquired late excretory phases and performed dynamic contrasted enhanced imaging at least 5 min after injection. Messina et al. (32) evaluated MRI performance to assess the risk of UTUC invasiveness including all sequences (T1–T2, DCE, and DWI).

### DCE without excretory phase

#### Diagnosis

The diagnostic performance of DCE without the excretory phase was studied in three studies (28, 40, 42) (**Table 2**). Pooled diagnostic performances showed 92.5% [95% IC 88.6%–95.2%] sensitivity, 76.7% [95% IC 58.5%–88.4%] specificity, and 91.1% [95% IC 86.3%–94.4%] accuracy (**Figure 2**).

#### Staging

The staging performances varied between the two studies (28, 42). Sensitivity varied between 83% and 88% sensitivity and 78% and 100% specificity for ≥T3b and ≤T3a tumor discrimination and 65% to 78% and specificity between 75% and 82% for ≥T3 and ≤T2 tumor discrimination.

### DCE with excretory phase

#### Diagnosis

Diagnosis performance could not be estimated because these three studies (28, 30, 31) evaluated segments or regions but no patient diagnosis.

The sensitivity for regional tumor detection was between 62% and 86%, and the specificity was between 80% and 97%.

Studies (30, 31) have shown that interpretation can be influenced by the quality of acquisition, suboptimal distention, and poor opacification of the collecting system or ureters.

### Diffusion weighted MRI

Some studies have reported the diagnostic performance of DWI for T categorization (28, 40).

#### Diagnosis

A total of six studies evaluated tumor detection performance (28, 29, 38–40, 42).

Pooled diagnostic performances showed 93.2% [95% IC 90.7%–95.1%] sensitivity, 84.2% [95% IC 76.3%–89.8%] specificity, and 90.1% [95% IC 86.6%–92.8%] accuracy (**Figure 2**). The pooled sensitivity was very high with a short confidence interval with data from five studies (28, 34, 38, 42) (**Figure 2**). The interobserver agreement κ was excellent, ranging from 0.801 to 0.934. The pooled specificity had a dispersed distribution, but the b-value seemed to change the specificity (38). DWI acquisition was performed with a *b-*value ranging from 400s/mm^2^ to 1,500 s/mm^2^, but DWI acquisition could impact the performance. Wu et al. (38) evaluated the performance of DWI with b-value of 500s/mm^2^ or 1,500 s/mm^2^. A high *b-*value reduced specificity but did not change sensitivity. Wu et al. (38) suggested that if a higher *b-*value signal is used, the intensity of non-malignant tissues decreases faster than those of malignant tissues, thus improving the ability to differentiate them.

#### Staging

Staging performances were interesting, with 81%–88% sensitivity and 96%–100% specificity for ≥T3b and ≤T3a tumor discrimination (28, 42). The performance for ≥T3 and ≤T2 tumor discrimination was lower, with 58%–80% sensitivity and 77%–93% specificity (28, 42).

#### Apparent diffusion coefficient

Six studies (33, 34, 36, 37, 41, 42) suggested that ADC could be a prognostic marker. Statistically, the mean ADC value of malignant lesions was significantly lower than that of both normal renal parenchyma and benign lesions (28, 33). Uchida et al. (36) demonstrated that lesions with a lower ADC value have a higher risk of developing metastasis. Yoshida et al. (41) demonstrated a significant inverse correlations of ADC with the histological grade and Ki-67 labeling index. Shebel et al. (34) defined the most significant cut off value of 1.5 x 10^3^ mm^2^/s to find the highest sensitivity and specificity of 79% and 82%, respectively for discriminating inflammatory lesions from urothelial tumors.

### Performance comparisons

Pooled performance comparisons were not possible due to study heterogeneity and the lack of consistent comparators between studies. Numerous studies have compared the performance of different MRI sequences.

#### MRI diagnosis

Standard acquisition was performed in only seven studies (28, 32, 34, 38, 40, 42); no study performed this acquisition alone, and it was always compared with DCE and/or DWI. Diagnosis performance were lower for standard acquisition in all studies, it was statistically significant in three studies (34, 38, 40).

Akita et al. (28) and Yoshida et al. (42) evaluated the diagnosis performance of standard acquisition against standard acquisition with DCE without excretory phase or standard acquisition with DWI in patient with confirmed UUTT. The tumor detection rate was not statistically different between the acquisitions (88% to 98%). In patient populations, including patients with suspected UUTT, Yoshida et al. (40) showed that the DWI sensitivity and accuracy (92% and 93%, respectively) were significantly greater than those of standard acquisition (69% and 80%, respectively), and the specificity was higher (96% *vs*. 89%), but without statistical significance. Likewise, Shebel et al. (34) found that DWI sensitivity (94%), specificity (77%), positive (92%) and negative predictive values (83%), and total accuracy (88%) in lesion detection were significantly higher (p <0.01) than those of standard acquisition (T1W + T2W) (86%, 77%, 91.6%, 66.6%, and 84%, respectively).

The diagnosis ability of DWI and DCE without the excretory phase was not markedly different (40), but Wu et al. (39) showed an increase in sensitivity for tumor diagnosis by adding DWI.

#### MRI staging

Akita et al. (28) and Yoshida et al. (42) evaluated the performances to discriminate T3 or higher tumors from T2 or lower tumors and ≤T3a *vs* ≥ T3b with differences sequences. There were no significant differences between sequences, except for the accuracy to discriminate ≤T3a *vs*. ≥T3b tumors, which was significantly better for standard acquisition with DWI (88% sensitivity and 96% specificity) than for standard acquisition alone (76% sensitivity and 74% specificity) (28).

Interestingly, less-experienced radiologist readers can increase their diagnostic performance by using additional sequences. For example, T2W plus DWI improved the accuracy and AUC results, while DCE without excretory phase improved the specificity, accuracy, and AUC for discriminating ≥T3b *vs* ≤T3a tumors (42). Interobserver agreement regarding the T categorization was excellent for standard acquisition with DWI (κ = 1) and DCE without the excretory phase (κ = 0.85), while it was good (κ = 0.69) for standard acquisition alone (28).

Messina et al. (32) evaluated the multiparametric MRI to assess the risk of UUTT muscle invasiveness and infiltration of perivisceral fat tissue. They used T2W imaging, T2W with fat saturation, DWI, ADC, and DCE with an excretory phase. The results are promising, with 81% and 95% sensitivity for less experienced (5 years) and more experienced (15 years) readers, respectively, and 61%–71% specificity and 72%–85% accuracy for muscle infiltration. For the infiltration of perivisceral fat tissue, the sensitivity, specificity, and accuracy were 78%–87%, 87%–93%, and 85%–92%, respectively.

### Where MRI fails?

False-positive and negative diagnoses using MRI were collected for each study (**Table 3**). Regardless of the sequence, MRI failed to detect carcinoma *in situ*. Due to section thickness and the intersection gap, small lesions were missed, with a cut-off range of 3 mm–5 mm (29, 33, 42).

**Table 3:** Where MRI failed?

Study	False negative	False positive
T1W + T2W		
Wu et al. (2013) (38) Imaging of upper urinary tract cancer: using conventional MRI and diffusion-weighted MRI with different b values		Non-specific inflammation Urothelial hyperplasia Ureteral papilloma
CE WITH excretory phase		
Lee et al. (2010) (30) Magnetic resonance urography versus retrograde pyelography/ureteroscopy for the exclusion of upper urinary tract malignancy		Ureteral stents *in situ* demonstrated ureteral wall enhancement, a small ureteral neoplasm could not be definitively excluded.
Martingano et al. (2013) (31) Magnetic resonance urography *vs* computed tomography urography in the evaluation of patients with hematuria	Small lesion	Filling defects Movement artefacts mimicking wall thickening
Takahashi et al. (2009) (35) Gadolinium Enhanced Magnetic Resonance Urography for Upper Urinary Tract Malignancy	Small renal pelvis lesion <4 mm Carcinoma in situ	Ureteral stone causing ureteral wall thickening Inflammatory ureteral stricture
T1W + T2W + DWI		
Akita et al. (2018) (29) Performance of DWI post-CT urography for the diagnosis of upper tract urothelial carcinoma: Comparison with selective urine cytology sampling	Ureter small papillary tumor <5 mm Carcinoma *in situ* Renal pelvis small papillary tumor	Inflammation Benign urinary tract wall thickening High signal intensity in renal papilla on DWI
Roy et al. (2015) (33) DWI in the Etiologic Diagnosis of Excretory Upper Urinary Tract Lesions: Can It Help in Differentiating Benign From Malignant Tumors?	Carcinoma *in situ* Small lesion <3 mm	
Wu et al. (2013) (39) Imaging of upper urinary tract cancer: using conventional MRI and diffusion-weighted MRI with different b values		Non-specific inflammation Urothelial hyperplasia Ureteral papilloma
Yoshida et al. (2010) (40) Usefulness of DWI in Diagnosis of Upper Urinary Tract Cancer	Carcinoma *in situ* Papillary ureteral cancer in the vesicoureteral junction (3 mm) Renal pelvic cancer with congested renal parenchyma	Ureteral inflammation Ureteral stenosis
Yoshida et al. (2017) (42) The value of adding DWI for tumor detection and preoperative staging in renal pelvic carcinoma for the reader’s experience	Small lesion Diffuse thin lesion	
CTU		
Akita et al. (2018) (29) Performance of DWI post-CT urography for the diagnosis of upper tract urothelial carcinoma: Comparison with selective urine cytology sampling	Ureter small papillary tumor Carcinoma *in situ*	Inflammation Benign urinary tract wall thickening Fibrosis Endometriosis Amyloidosis
Martingano et al. (2013) (31) Magnetic resonance urography *vs* computed tomography urography in the evaluation of patients with hematuria	Insufficient contrast medium excretion in obstructed patients	

CTU, computed tomography urography; DWI, diffusion weighted imaging; MRI, Magnetic resonance imaging; CE MRI, contrast enhanced magnetic resonance imaging; T2W, T2 weighted; T1W, T1 weighted.

Non-specific inflammation has always been considered as a suspicious lesion (29, 38, 40). ADC acquisition can also differentiate inflammation from malignant lesions (34). Movement artifacts pose an important challenge in MRI interpretation (31). It can mimic wall thickening.

### Performing an upper urinary tract MRI: conditions applied in selected studies

Hematuria changes the signal with an increasing signal on DWI with a low ADC value. It is preferable to postpone the examination for 2 weeks after an episode of gross hematuria (33).

The accuracy of MRI in detecting UUTT tends to be lower in patients with ureteral stents or nephrostomy tubes (35), which can cause urinary tract wall thickening that could mimic a urinary tumor (30, 35). It is not recommended to evaluate patients with ureteral stents or nephrostomy tubes using MRI alone.

Ureter and collecting system distention seems to improve imaging analysis (31). Therefore, six studies injected furosemide if no urinary dilatation was observed on the first sequence (30, 31, 33, 35). It is recommended to inject furosemide if no urinary dilatation is observed.

Some studies (31, 42) suppressed the gastrointestinal peristalsis. This study aimed to control motion artifacts from gastrointestinal exercise with hyoscine-N-butylbromide administration.

Ureteroscopy in the previous days of imaging examination caused peri-urethral infiltration, and it is preferable to postpone the examination (29, 33).

### Methodological quality: risk of bias assessment

**Figure 3:**
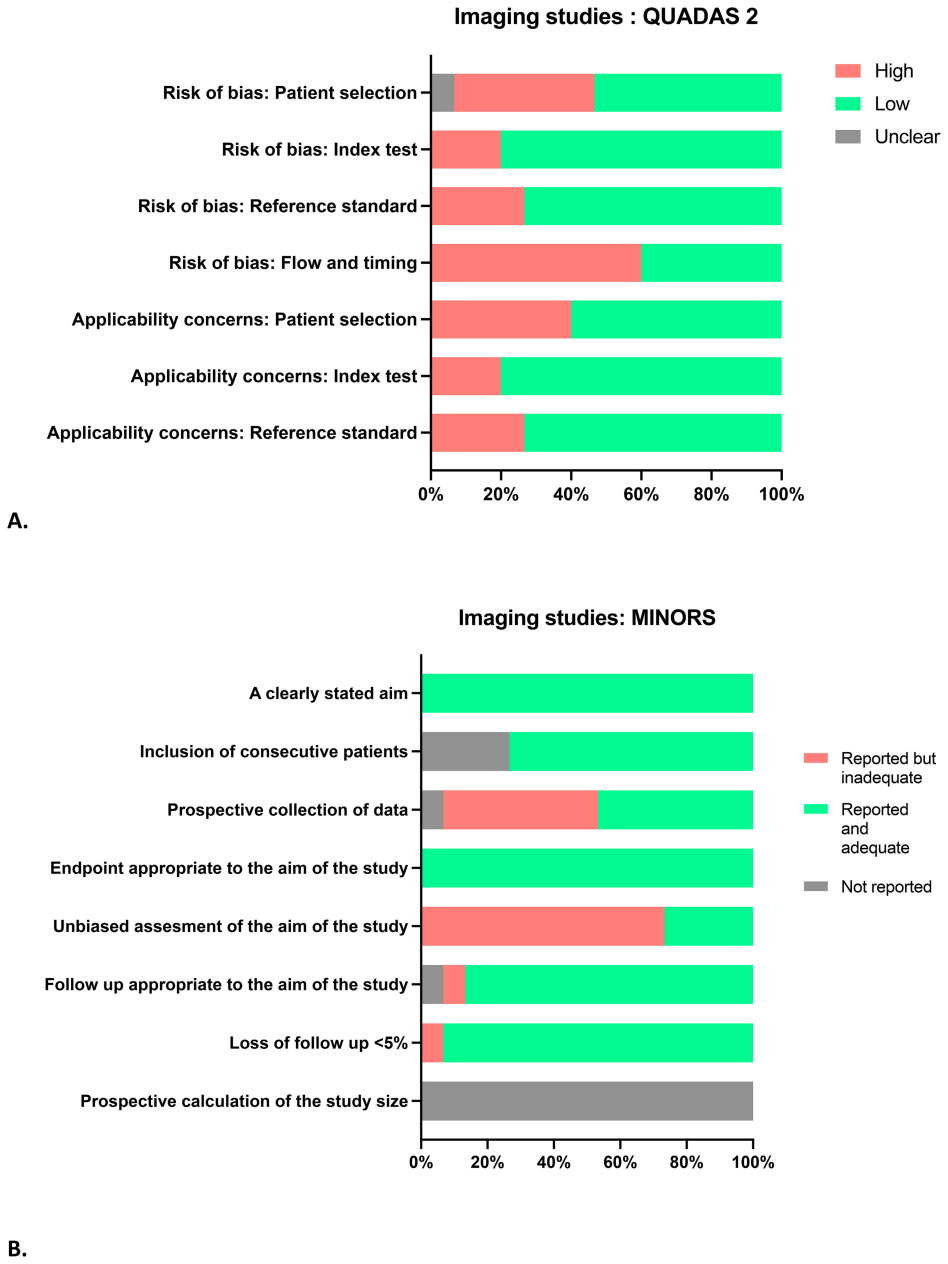
Methodological quality: risk of bias assessment QUADAS-2 **(A)** and MINORS **(B)**.

The risk of bias assessment is presented in **Figure 3**. Sixty percent (n = 9) of the studies were retrospective. Only three studies were considered to have a low risk of bias in all domains. A high risk of bias was deemed in 27% (n = 4) of the studies due to the lack of control cases; they selected patients with UUTT only. Additionally, 20% (n = 3) of the studies informed radiologists of the presence of at least one UUT lesion in each examination, which introduced bias. In 60% (n = 9) of the included studies, there was a high risk of bias for flow and timing due to unclear intervals between the index test and reference standard. Different reference standards were used in the studies (nephroureterectomy, biopsy, imaging).

## Discussion

The diagnosis and follow-up of UUTT must be improved to be more accurate and less invasive. Differentiating T1 or lower stage tumors from T2 and higher stage tumors represents a new challenge in the management of UUTT patients. There is an exciting prospect that MRI could increase the accuracy of the diagnosis and the staging of the UUTT without general anesthesia, radiation or use of nephrotoxic contrast. CTU is the reference examination for detecting UUTT. In contrast to CTU, MRI is the only test available for the diagnosis of muscle invasion and appears to be reliable, with 85% accuracy for MRI, versus 43% misclassification for biopsy (16, 32).

This is the first systematic review to include all currently available studies that evaluated the diagnostic performance of UUT MRI. Three key concepts are highlighted. First, the available literature data are scarce, and only one study has been published in the past 6 years. Messina et al. (32) highlights UUT MRI’s promising results; MRI could be used to diagnose UUTT perivisceral fat infiltration and especially muscle-layer involvement. Our study encourages further research.

Second, the available studies included different populations and had heterogeneous designs, such as different MRI protocols, yielding highly variable results. Third, only study evaluated the ability of MRI to differentiate between <T2 and ≥T2 stage tumors, but showed interesting results. Finally, contrary to reference imaging (CTU), MRI can be used to assess prognosis and tissue structure. MRI is the only imaging modality that can detect muscle infiltration.

We have shown that MRI with functional non-contrast imaging sequences provides information beyond anatomical structure, including tissular structure and prognosis.

The excretory phase is of interest for assessing tumor obstruction and endoluminal development. Studies have suggested that DWI can improve specificity (29, 38).

It is interesting to note that despite the fact that DWI sequences are not anatomical and are difficult to read, less-experienced readers can increased their diagnostic performance by adding DWI sequences (42) and radiologist confidence (39).

Functional MRI, such as DWI and ADC, combined with DCE and the excretory phase, represents interesting perspectives for UUT evaluation, with possibilities for diagnosis, staging, and prognosis evaluation.

Based on this analysis, it seems essential that future studies on UUT MRI include T1W, T2W, DCE with excretory phase, DWI with high b-value (>800 s/mm^2^), and ADC analysis in the imaging protocol. All these sequences should be performed with a strong consensus at the conference of the French Society of Genitourinary Imaging (44).

Thanks to well-conducted studies, we hope to establish an Upper Urinary Tract Imaging-Reporting and Data System (UUTI-RADS) score for predicting muscle invasion and tumor stage. Once the UUTI-RADS is established, radiomics data may be used to predict prognosis and response to chemotherapy.

### Limitations

This study has some limitations. First, the small sample size of the studies influences the accuracy of the results and makes it difficult to extract reliable performance parameters. Many of the included studies were retrospective. Some studies informed radiologists of the presence of at least one UUTT lesion on each examination so the blinding is flowed. The reference standard varies between patients and studies. For a perfect methodology, only surgical specimens should be used as the reference standard. The populations differed with variability in the clinical presentation of patients, selection of patients from the included studies, and limit analysis. MRI sequences are heterogeneous, making it difficult to compare the results. Finally, the major limitation of this review is the lack of high-level evidence evaluating MRI in the diagnosis of UUT,T such as the heterogeneity of data, the lack of prospective studies, and the standardization of imaging.

## Conclusion

This review has shown a lack of evidence regarding the usefulness of MRI and its diagnostic performance for UUTT. However, these results and the addition of DWI sequences are encouraging and suggest an interesting perspective for UUTT diagnosis, treatment, and management. This review should encourage new prospective studies and facilitate their design by reviewing the current data available and identifying the modalities of performing MRI for UUT assessment.

A large multicenter study evaluating the diagnostic performance of UUT MRI with a predefined standard acquisition (including T1W, T2W, DWI, and DCE with excretory phase acquisition) compared to the pathology report of radical nephroureterectomy or long-term follow-up by medical imaging will be proposed, and we hope to establish an Upper Urinary Tract Imaging-Reporting and Data System (UUTI-RADS) score for predicting muscle invasion and tumor stage.

## Data Availability

The original contributions presented in the study are included in the article/**Supplementary Material**. Further inquiries can be directed to the corresponding author.
