# Supplementary material for: The role of MRI in detection and staging of upper urinary tract cancer: a systematic review of the literature
Source: Front Oncol. 2026 Feb 19;16:1563391. doi: 10.3389/fonc.2026.1563391 (PMC12960106; doi:10.3389/fonc.2026.1563391)
Supplement: Supplementary file 2 [file Table2.docx]

**Supplementary Table 2 :** Details of search terms

| **Search Strategy for MEDLINE (PubMed)** | |
| --- | --- |
| **Key words** | (("ureter"[MeSH Terms] OR "ureteral diseases"[MeSH Terms] OR (("upper"[All Fields] OR "uppers"[All Fields]) AND ("urinary tract"[MeSH Terms] OR ("urinary"[All Fields] AND "tract"[All Fields]) OR "urinary tract"[All Fields])) OR (("upper"[All Fields] OR "uppers"[All Fields]) AND ("urinary tract"[MeSH Terms] OR ("urinary"[All Fields] AND "tract"[All Fields]) OR "urinary tract"[All Fields]) AND ("carcinoma, transitional cell"[MeSH Terms] OR ("carcinoma"[All Fields] AND "transitional"[All Fields] AND "cell"[All Fields]) OR "transitional cell carcinoma"[All Fields] OR ("urothelial"[All Fields] AND "carcinoma"[All Fields]) OR "urothelial carcinoma"[All Fields])) OR ("ureter"[MeSH Terms] OR "ureter"[All Fields] OR "ureters"[All Fields] OR "ureter s"[All Fields] OR "uretic"[All Fields]) OR ("kidney pelvis"[MeSH Terms] OR ("kidney"[All Fields] AND "pelvis"[All Fields]) OR "kidney pelvis"[All Fields] OR ("renal"[All Fields] AND "pelvis"[All Fields]) OR "renal pelvis"[All Fields]) OR ("carcinoma, transitional cell"[MeSH Terms] OR ("carcinoma"[All Fields] AND "transitional"[All Fields] AND "cell"[All Fields]) OR "transitional cell carcinoma"[All Fields] OR ("urothelial"[All Fields] AND "carcinoma"[All Fields]) OR "urothelial carcinoma"[All Fields])) AND ("diffusion magnetic resonance imaging"[MeSH Terms] OR "diffusion tensor imaging"[MeSH Terms] OR "magnetic resonance imaging"[MeSH Terms] OR ("magnetic resonance imaging"[MeSH Terms] OR ("magnetic"[All Fields] AND "resonance"[All Fields] AND "imaging"[All Fields]) OR "magnetic resonance imaging"[All Fields] OR "mri"[All Fields]) OR (("diffusable"[All Fields] OR "diffusant"[All Fields] OR "diffusants"[All Fields] OR "diffuse"[All Fields] OR "diffusely"[All Fields] OR "diffuses"[All Fields] OR "diffusibility"[All Fields] OR "diffusible"[All Fields] OR "diffusion"[MeSH Terms] OR "diffusion"[All Fields] OR "diffused"[All Fields] OR "diffusing"[All Fields] OR "diffusions"[All Fields] OR "diffusive"[All Fields] OR "diffusively"[All Fields] OR "diffusivities"[All Fields] OR "diffusivity"[All Fields]) AND ("weight s"[All Fields] OR "weighted"[All Fields] OR "weighting"[All Fields] OR "weightings"[All Fields] OR "weights and measures"[MeSH Terms] OR ("weights"[All Fields] AND "measures"[All Fields]) OR "weights and measures"[All Fields] OR "weight"[All Fields] OR "body weight"[MeSH Terms] OR ("body"[All Fields] AND "weight"[All Fields]) OR "body weight"[All Fields] OR "weights"[All Fields])) OR ("magnetic resonance imaging"[MeSH Terms] OR ("magnetic"[All Fields] AND "resonance"[All Fields] AND "imaging"[All Fields]) OR "magnetic resonance imaging"[All Fields]) OR ("diffusion magnetic resonance imaging"[MeSH Terms] OR ("diffusion"[All Fields] AND "magnetic"[All Fields] AND "resonance"[All Fields] AND "imaging"[All Fields]) OR "diffusion magnetic resonance imaging"[All Fields]) OR (("diffusable"[All Fields] OR "diffusant"[All Fields] OR "diffusants"[All Fields] OR "diffuse"[All Fields] OR "diffusely"[All Fields] OR "diffuses"[All Fields] OR "diffusibility"[All Fields] OR "diffusible"[All Fields] OR "diffusion"[MeSH Terms] OR "diffusion"[All Fields] OR "diffused"[All Fields] OR "diffusing"[All Fields] OR "diffusions"[All Fields] OR "diffusive"[All Fields] OR "diffusively"[All Fields] OR "diffusivities"[All Fields] OR "diffusivity"[All Fields]) AND ("magnet s"[All Fields] OR "magnetical"[All Fields] OR "magnetically"[All Fields] OR "magnetics"[MeSH Terms] OR "magnetics"[All Fields] OR "magnetic"[All Fields] OR "magnetisation"[All Fields] OR "magnetisations"[All Fields] OR "magnetised"[All Fields] OR "magnetism"[All Fields] OR "magnetisms"[All Fields] OR "magnetization"[All Fields] OR "magnetizations"[All Fields] OR "magnetize"[All Fields] OR "magnetized"[All Fields] OR "magnetizing"[All Fields] OR "magnets"[MeSH Terms] OR "magnets"[All Fields] OR "magnet"[All Fields]) AND ("image"[All Fields] OR "image s"[All Fields] OR "imaged"[All Fields] OR "imager"[All Fields] OR "imager s"[All Fields] OR "imagers"[All Fields] OR "images"[All Fields] OR "imaging"[All Fields] OR "imaging s"[All Fields] OR "imagings"[All Fields])) OR "dwi"[All Fields] OR ("arch dis child"[Journal] OR "acta dermatovenerol croat"[Journal] OR "adc"[All Fields]) OR ("apparent"[All Fields] AND ("diffusable"[All Fields] OR "diffusant"[All Fields] OR "diffusants"[All Fields] OR "diffuse"[All Fields] OR "diffusely"[All Fields] OR "diffuses"[All Fields] OR "diffusibility"[All Fields] OR "diffusible"[All Fields] OR "diffusion"[MeSH Terms] OR "diffusion"[All Fields] OR "diffused"[All Fields] OR "diffusing"[All Fields] OR "diffusions"[All Fields] OR "diffusive"[All Fields] OR "diffusively"[All Fields] OR "diffusivities"[All Fields] OR "diffusivity"[All Fields]) AND ("coefficiencies"[All Fields] OR "coefficiency"[All Fields] OR "coefficient"[All Fields] OR "coefficient s"[All Fields] OR "coefficients"[All Fields])))) |
| **Publication period** | (2000:2024[pdat])) |
| **Search filters** | AND Full text ((fft[Filter]); AND english[Filter]) |
|  |  |
| **Search Strategy for Embase** | |
| **Key words** | ('mri'/exp OR 'mri' OR 'magnetic resonance imaging' OR 'magnetic resonance urography' OR 'diffusion magnetic imaging' OR 'DWI' OR 'diffusion weighted imaging') AND ('upper urinary tract urothelial carcinoma'/exp OR 'upper urinary tract urothelial carcinoma' OR 'ureteral neoplasm' OR 'upper urinary tract' OR 'ureter disease' OR 'ureter' OR ‘renal pelvis’) |
| **Publication period** | 01-01-2000]/sd NOT [08-01-2024]/sd |
| **Search filters** | [english]/lim NOT ('nonhuman'/de OR 'case report'/de OR 'medical record review'/de OR 'meta analysis'/de OR 'systematic review'/de) |
|  |  |
| **Search Strategy for The Cochrane Library (Cochrane Central Register of Controlled Trials)** | |
| **Key words** | Upper urinary tract* in Title Abstract Keyword OR ureter* in Title Abstract Keyword OR renal pelvis* in Title Abstract Keyword AND magnetic resonance imaging* in Title Abstract Keyword OR apparent diffusion coefficient* in Title Abstract Keyword OR diffusion weighted imaging* in Title Abstract Keyword - (Word variations have been searched) |
| **Publication period** | 01/01/2000 to 08/01/2024 |
| **Search filters** | ∅ |
