# Supplementary material for: The role of MRI in detection and staging of upper urinary tract cancer: a systematic review of the literature
Source: Front Oncol. 2026 Feb 19;16:1563391. doi: 10.3389/fonc.2026.1563391 (PMC12960106; doi:10.3389/fonc.2026.1563391)
Supplement: Supplementary file 3 [file Table3.docx]

**Supplementary Table 3** : standardized form used for data extraction

| General data of the article | | | | Study design characteristics | | | | | Population characteristic | | | | | | | | | | Radiologist and pathologist characteristics | | | | | | MRI characteristic | | | | | | | | | | Result | | | | | |
| --- | --- | --- | --- | --- | --- | --- | --- | --- | --- | --- | --- | --- | --- | --- | --- | --- | --- | --- | --- | --- | --- | --- | --- | --- | --- | --- | --- | --- | --- | --- | --- | --- | --- | --- | --- | --- | --- | --- | --- | --- |
|  |  |  |  |  |  |  |  |  |  |  |  | Tumor stage | | | | | |  | Radiologist | | | Pathologist | | |  |  |  |  |  |  |  |  |  |  |  |  |  |  |  |  |
| Title | Authors | Year of publication | Journal | Prospective/retrospective | Unicentric/multicentric | Consecutive/non consecutive enrolment | Duration of patient recruitment | Reference standard | Total Number | Carcinoma number | No carcinoma number | Cis number | Ta number | T1 number | T2 number | T3 Number | T4 Number | Number of patient with renal failure | Number of radiologist | Experience of radiologist | Patient information for the interpretation of the exam | Number of pathologist | Experience of pathologist | Patient information for the interpretation of the exam | Magnet field strength | Scanner model | Manufecturer | Coil | T1W | T2W | CE | DWI | DCE | Set analysis | Accuracy | Sensitivity | Specificity | Inter reader agreement | False negative explanation | False positive explanation |
|  |  |  |  |  |  |  |  |  |  |  |  |  |  |  |  |  |  |  |  |  |  |  |  |  |  |  |  |  |  |  |  |  |  |  |  |  |  |  |  |  |
|  |  |  |  |  |  |  |  |  |  |  |  |  |  |  |  |  |  |  |  |  |  |  |  |  |  |  |  |  |  |  |  |  |  |  |  |  |  |  |  |  |
|  |  |  |  |  |  |  |  |  |  |  |  |  |  |  |  |  |  |  |  |  |  |  |  |  |  |  |  |  |  |  |  |  |  |  |  |  |  |  |  |  |
|  |  |  |  |  |  |  |  |  |  |  |  |  |  |  |  |  |  |  |  |  |  |  |  |  |  |  |  |  |  |  |  |  |  |  |  |  |  |  |  |  |
|  |  |  |  |  |  |  |  |  |  |  |  |  |  |  |  |  |  |  |  |  |  |  |  |  |  |  |  |  |  |  |  |  |  |  |  |  |  |  |  |  |
|  |  |  |  |  |  |  |  |  |  |  |  |  |  |  |  |  |  |  |  |  |  |  |  |  |  |  |  |  |  |  |  |  |  |  |  |  |  |  |  |  |
|  |  |  |  |  |  |  |  |  |  |  |  |  |  |  |  |  |  |  |  |  |  |  |  |  |  |  |  |  |  |  |  |  |  |  |  |  |  |  |  |  |
|  |  |  |  |  |  |  |  |  |  |  |  |  |  |  |  |  |  |  |  |  |  |  |  |  |  |  |  |  |  |  |  |  |  |  |  |  |  |  |  |  |
|  |  |  |  |  |  |  |  |  |  |  |  |  |  |  |  |  |  |  |  |  |  |  |  |  |  |  |  |  |  |  |  |  |  |  |  |  |  |  |  |  |
|  |  |  |  |  |  |  |  |  |  |  |  |  |  |  |  |  |  |  |  |  |  |  |  |  |  |  |  |  |  |  |  |  |  |  |  |  |  |  |  |  |
|  |  |  |  |  |  |  |  |  |  |  |  |  |  |  |  |  |  |  |  |  |  |  |  |  |  |  |  |  |  |  |  |  |  |  |  |  |  |  |  |  |
|  |  |  |  |  |  |  |  |  |  |  |  |  |  |  |  |  |  |  |  |  |  |  |  |  |  |  |  |  |  |  |  |  |  |  |  |  |  |  |  |  |
